# ADAPTS: An Intelligent Sustainable Conceptual Framework for Engineering Projects

**DOI:** 10.3390/s20061553

**Published:** 2020-03-11

**Authors:** Amalia Luque, Ana De Las Heras, María Jesús Ávila-Gutiérrez, Francisco Zamora-Polo

**Affiliations:** Dpto. Ingeniería del Diseño. Escuela Politécnica Superior. Universidad de Sevilla. Virgen de África, 7. 41011 Sevilla, Spain; adelasheras@us.es (A.D.L.H.); mavila@us.es (M.J.Á.-G.); fzpolo@us.es (F.Z.-P.)

**Keywords:** conceptual framework, sensors, approaches, tools, data, application, project engineering, LCA, SDG 9, SDG 11

## Abstract

This paper presents a conceptual framework for the optimization of environmental sustainability in engineering projects, both for products and industrial facilities or processes. The main objective of this work is to propose a conceptual framework to help researchers to approach optimization under the criteria of sustainability of engineering projects, making use of current Machine Learning techniques. For the development of this conceptual framework, a bibliographic search has been carried out on the Web of Science. From the selected documents and through a hermeneutic procedure the texts have been analyzed and the conceptual framework has been carried out. A graphic representation pyramid shape is shown to clearly define the variables of the proposed conceptual framework and their relationships. The conceptual framework consists of 5 dimensions; its acronym is ADAPTS. In the base are: (1) the Application to which it is intended, (2) the available DAta, (3) the APproach under which it is operated, and (4) the machine learning Tool used. At the top of the pyramid, (5) the necessary Sensing. A study case is proposed to show its applicability. This work is part of a broader line of research, in terms of optimization under sustainability criteria.

## 1. Introduction

In recent years concern for sustainability has grown, becoming one of the main problems worldwide [1,2]. The 2030 Agenda for Sustainable Development sets out 17 Sustainable Development Goals with 169 goals of an integrated and indivisible nature that cover the economic, social and environmental spheres. An example of the importance that environmental sustainability has at the moment is that several of the Sustainable Development Goals are directly related to the concepts of sustainability [3,4].

Many of the interpretations of what sustainable development should be agree that, in order to achieve this, the policies and actions to achieve economic growth must respect the environment and also be socially equitable to achieve economic growth: it is the model of Triple E [5,6].

The main objective of this work is to propose a conceptual framework to help researchers to approach optimization under the criteria of sustainability of engineering projects, making use of current Machine Learning techniques.

Likewise, the growing development in collaborative methodologies such as BIM (a methodology that integrates a growing number of disciplines and processes involved in the life cycle of a project), is something to consider. The sixth dimension of BIM is not only about energy saving and sustainable design (although they are the most recognized aspects), but also about the concept of value engineering, which consists in the optimization of construction systems, structures and facilities, so that with a few key modifications in strategic items or in certain systems or equipment it could be possible to obtain a significant reduction in costs, in the construction phase and / or in the exploitation phase, without losing the essence of the project. In a project that incorporates the sixth dimension of the BIM, analytical models are generated to perform analysis, calculations and simulations in order to improve the quality of the project [7,8].

For the optimization of sustainability throughout the entire life cycle of a particular product, process or industrial installation, it is necessary to use some methodology for quantification and evaluation of sustainability.

**Figure 1:**
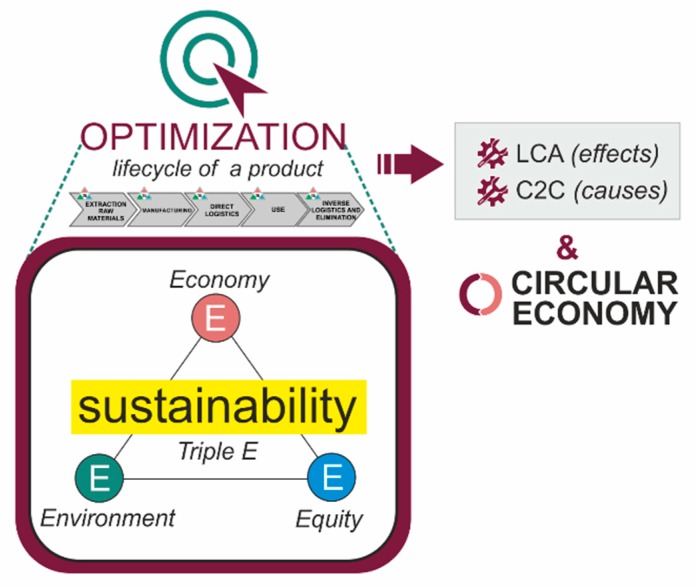
Sustainability, Triple Bottom Line and Optimization. LCA/C2C/Circular Economy.

There are techniques aimed primarily at controlling effects (for example, life cycle analysis-LCA) [9,10] and techniques more focused on eliminating the causes (among which is Cradle to Cradle-C2C) [11,12].We can also find the circular economy approach, which proposes a new model of society that uses and optimizes stocks and flows of materials, energy and waste, with the aim of maximizing the efficiency of resource use [13,14] (Figure 1).

However, these techniques, more oriented to modify the causes of sustainability inefficiencies, do not provide rigorous quantitative techniques for their evaluation (although they can be very useful in the decision-making process) [15,16]. That is why it seems appropriate to deepen the study of the LCA as a tool for evaluating potential impacts.

Life Cycle Analysis (LCA) is an objective process that allows evaluating the environmental loads associated with a product, process or activity, identifying and quantifying both the use of matter and energy and the emissions to the environment, to determine the impact of that use of resources and those emissions [17,18].

On the other hand, industry is currently immersed in an evolution, consisting of its digital transformation [19,20]. To understand the keys to this transformation it is necessary to know the available tools and other emerging ones of new application. The term Industry 4.0. is a recent term, which consists in the use of new technologies and the integration with some other evolved ones, to achieve the total or partial digitalization of the current productive models, or to create new models that allow a substantial reduction of the terms in all the phases of the affected project or services and efficiency improvements of all kinds [21,22].

Among the solutions related to Industry 4.0 with more future are artificial intelligence and machine learning. Artificial intelligence techniques have evolved vertiginously recently [23], becoming a very useful tool to address complex problems in many different fields. These types of techniques have been applied to problems in the fields of sound classification [24], image processing [25,26], risk prevention [27,28], air conditioning [29,30], in estimation of project parameters [31,32], and in many other fields of research. We understand very important to deepen the study of possible fields of application of these tools.

**Figure 2:**
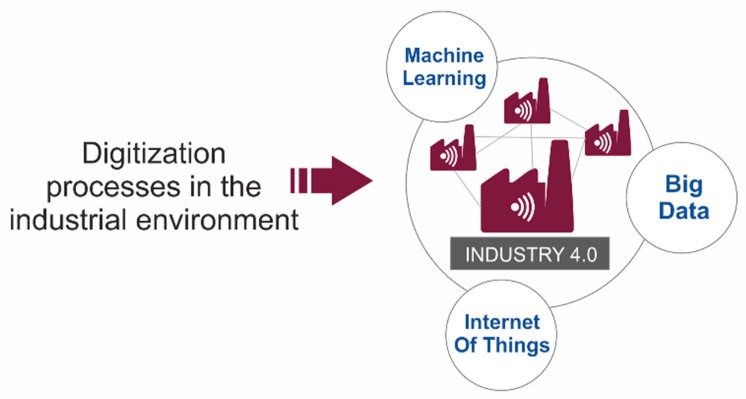
Three digitalization process.

One of the challenges is that machine learning algorithms need to have a large volume of data in order to be trained, validated and generalized. There are multiple technologies that are framed within Industry 4.0. However, we can circumscribe the processes of digitization of industrial environments around three elements (Figure 2):

- Collection of data from machines, warehouses and articles, achieved through the Internet of Things (IoT).
- Analysis and exploitation of this huge amount of data through big data and business intelligence (BI) techniques.
- Predictive analytics based on data through machine learning.

Information is of crucial importance on these three stages. Obtaining, storing and managing relevant data intelligently plays a decisive role in the so-called Industry 4.0. [33,34]. In this way, Big Data becomes the axis on which the rest of actions must pivot in terms of digitalization. However, the avalanche of data faced by organizations may pose a threat to the viability of the project.

In the first phase of industrial digitalization, companies need to capture in real time the maximum possible information, structured and accessible, about what is happening in their business. The development of intelligent sensors, capable of being located in multiple locations of the industrial processes, allows to capture a large number of parameters based on various indicators.

**Figure 3:**
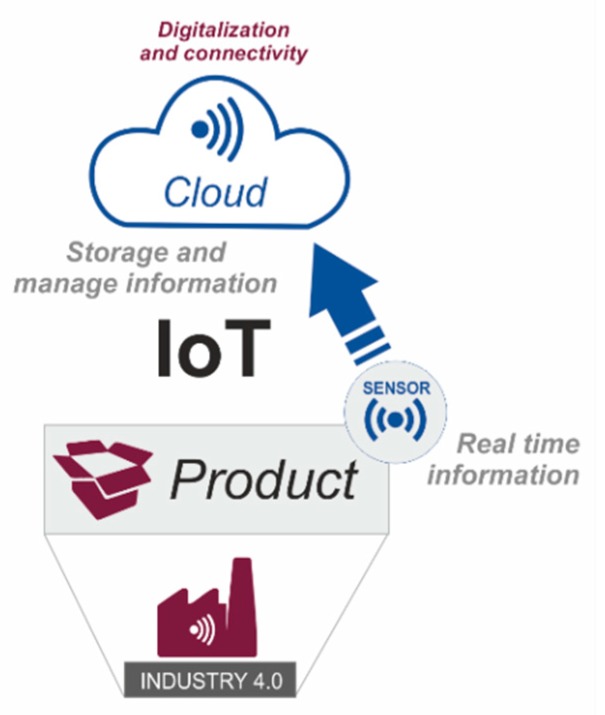
Sensors/Cloud/(connectivity)/ Storage (connectivity).

These systems are complemented with cloud technology (Figure 3). The emergence of cloud solutions allows companies to store and manage in real time the multiple measurements obtained from sensors. This process is what we call the Internet of things (IoT). There are a number of advantages inherent to IoT that could be taken advantage of immediately by the industry, such as the detection of possible failures, the forecast of wear of parts or the reconfiguration of parameters and calibrations. However, many professionals are still cautious about their actual application in the industry. In this sense, there is a widespread perception that the real industrial world is not so prepared for hyper connectivity [35,36].

It is necessary to rely on agents that cover this gap from analog systems to intelligent ones where the technology allows to connect the real world to communicate it to the cloud through the so-called cyber-physical systems. In any case, it is an unstoppable trend since several studies estimate that by 2020 there will be 212 billion devices connected with sensors in the world [37].

**Figure 4:**
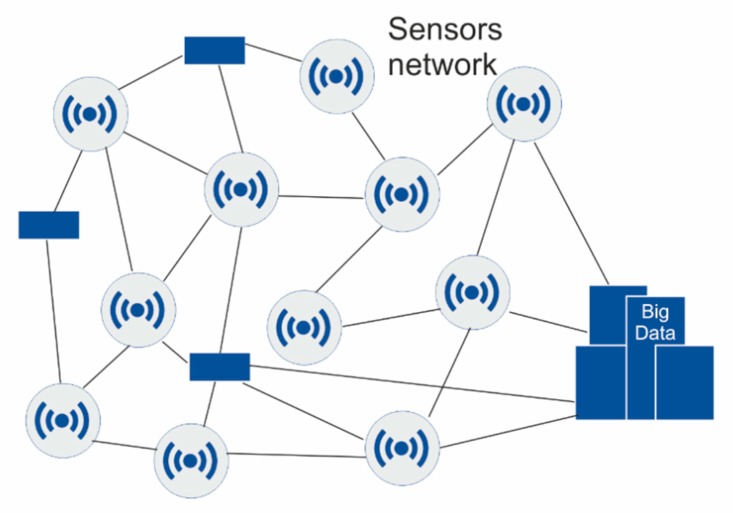
Sensors network.

The second phase consists in analyzing what is happening through tools that identify patterns and inefficiencies. Big data solutions allow the collection and systematized treatment of relevant data, obtained through sensorization, to make decisions that affect both the immediate future of organizations (to solve a breakdown or replace a part) and its development in the long term (change supplier, modify packaging or renew machinery) (Figure 4).

It is a very complex process due to two factors: the huge volume of data and the heterogeneity of the sources from where come the data.

In addition to this, we do not only want to know what it is that has happened or is happening in those moments, but what is going to happen in the near future. This is achieved by providing the manufacturing equipment of intelligent applications that make measurements along all processes by combining the historical data and predictive models. Machine learning can be then understood as the last phase of the evolution of Industry 4.0 [38,39].

The predictive analysis, based on artificial intelligence, requires the design and implementation of algorithms that learn to represent data and to detect trends. The objective of the system is to develop models of future behaviors from the data. In this sense, the quality and quantity of information it is essential to take the next steps towards the Fourth Industrial Revolution. As we see, this brings us back to Big Data.

At this point we will return to the objective approach to the optimization under the criteria of sustainability of engineering projects, making use of the current techniques of Machine Learning. We will focus on the evaluation of the sustainability using the technique of LCA, because it uses a few indicators that allow to quantify the environmental impacts [9,40].

Given that the techniques of artificial intelligence are a very useful tool for dealing with complex problems in diverse fields, it seems interesting to raise the possibility of using them as an aid in this goal. Therefore, it is essential to take account of the need to collect and make a systematic treatment of the relevant data, obtained through smart sensing.

**Figure 5:**
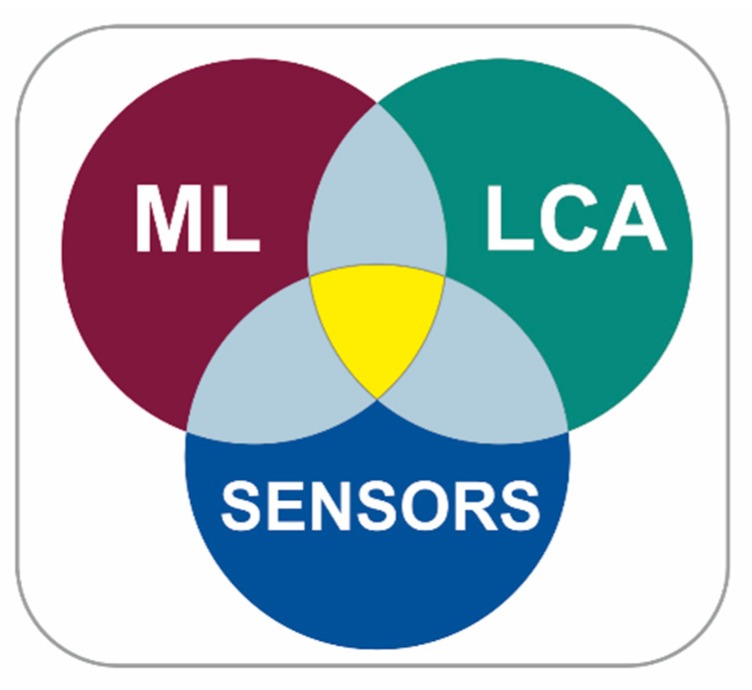
ML-LCA-SENSORS.

As far as we know, the sensorization of industrial plants and the treatment of data through machine learning algorithms is not yet sufficiently extended in the field of sustainability optimization. We detect a gap of opportunity (Figure 5) in the investigation of the state of the art, with the objective of proposing a conceptual framework that can help in the approach to this type of problems.

**Figure 6:**
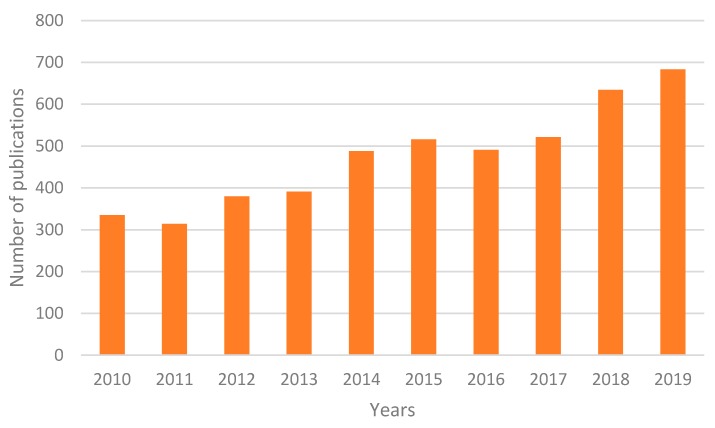
Number of published articles containing "conceptual framework" in the title indexed in Scopus. Source: Scopus (2020).

Conceptual frameworks are a very useful tool widely used for various applications. Figure 6 shows the evolution in the number of articles contained "conceptual framework" in the title indexed in the SCOPUS database over the last 10 years. As can be seen, there is a growing interest in the development of this type of work by the scientific community.

In [41] the use of portable sensors to provide an adaptation based on affection in Environmental Intelligence systems is considered, and a proposal for a conceptual design framework for games is presented. Zupančič et al. [42] defines a conceptual framework that considers human participation in mobile crowd detection systems and takes into account that users provide their opinions and other subjective data in addition to unprocessed detection data generated by your smart devices. Mordecai et al. [43] proposes a conceptual approach based on models to capture, explain and mitigate CPG, which improves the systems engineer’s ability to cope with CPGs, mitigate them by design and avoid decisions and wrong actions. González et al. [44] presents a new architecture based on OPC to implement automation systems dedicated to R&D and educational activities. The proposal is a new conceptual framework, structured in four functional layers where the various components are classified with the objective of promoting the systematic design and implementation of automation systems that involve OPC communication. Yoo et al. [45] describe a conceptual framework for exchanging closed cycle life cycle information for the service of products on the Internet of Things (IoT). The framework is based on the product-service ontology model and a standard IoT message type, Open Messaging Interface (O-MI) and Open Data Format (O-DF), which guarantees data communication. Varela et al. [46] states that human interaction environments (HIE) should be understood as any place where people carry out their daily lives, including their work, family life, leisure and social life, interacting with technology to improve or facilitate the experience. The integration of technology in these environments has been achieved in a disorderly and incompatible way, with devices that operate on isolated islands with artificial borders delimited by manufacturers. A framework is presented that constitutes an integral solution for the development of systems that require the integration and interoperation of devices and technologies in HIE. In this work a conceptual framework for the integration of artificial intelligence and life cycle assessment (LCA) will be proposed.

## 2. Methodology

The objective of this work is the development of a conceptual framework for the integration of artificial intelligence and LCA. A conceptual framework is a tool that allows the analysis and organization of information related to a field of knowledge. Thus, it is easier to carry out future research [47,48].

An important part of academic texts has to do with integrating concepts, ideas, arguments or theories of our discipline that allow us to fulfill the objectives of a writing work. Sometimes, these concepts and ideas work to briefly explain the object or theme of our text, but in other cases a more extensive conceptual framework must be developed to meet the requirements of the task.

A conceptual framework is a section of a text written in the academic field that details the theoretical models, concepts, arguments and ideas that have been developed in relation to a topic. The conceptual framework is generally oriented to define this object, describe its characteristics and explain possible processes associated with it. In some more extensive texts, the conceptual framework also works to recognize and describe “the state of the art”, that is, to point out the main theoretical lines in relation to this topic, in order to propose a new theoretical view that is considered relevant in relationship with the object.

All research needs to use concepts to be able to organize your data and perceive the relationships between them. Borsotti [49] suggests that scientific knowledge is entirely conceptual, since; ultimately, it is constituted by interrelated systems of concepts in different ways. Hence, to access the ideas of science, it is necessary to manage the concepts and languages of science. These concepts cannot cease to be subjective; they are necessarily conditioned by ideological positions and by evaluative positions that are logical assumptions of all knowledge. Borsotti adds, that "when you think about it, it is irremediable to resort to notions drawn from common language, generated in historical and social life, and that are loaded with ideological connotations and full of ambiguity and vagueness. Science cannot be managed with these concepts. It does not seek to be exact, but to be precise, in order to achieve the elaboration, the construction of unique concepts, that is, concepts whose intention and extension are as precise as possible." A concept is an abstraction obtained from reality and, therefore, its purpose is to simplify by summarizing a series of observations that can be classified under the same name.

The information that is integrated into the conceptual framework must be systematically organized so that it can be better understood. An important principle is to start from the most general to the most particular. A starting point can be the definition of the object or topic and then describe its characteristics, functions and indicate the parts that compose it or the associated concepts that are relevant.

The diagram representing the research topic or problem is sometimes called the conceptual framework, this diagram could be useful to analysis and interpret the results [47,48,50]. It is a visual scheme that represents the concept or idea. It is the way in which the work will be carried out and integrates the elements. It also influences the research problem as it is associated with the literature used. A part of this framework will offer a synopsis of the main points of the study. In addition, the diagram will show the central factors that influence the relationship of the primary variables, elements or constructions, as well as the hypothesis. After reading the literature of the corresponding area, it has to be shown what the theories state about it and support the relationship.

The first phases followed in the methodology for developing the conceptual framework include investigating the main variables or elements that correspond to those contextual factors that are related to the research work. The technique used in this initial stage for constructing the conceptual framework is the hermeneutics. In hermeneutics, the texts are read and analyzed in order to delve into them obtaining a better understanding of reality [51]. Although the origin of hermeneutics is associated with the study and interpretation of religious texts, its use has been extended to other disciplines such as pedagogy or philosophy [52]. With the expression hermeneutic circle, the relationship between the text and the context from which the research is revealed [50,52]. In our case, it has been successfully applied to the construction of a conceptual framework for the teaching of Sustainable Development Goals in Higher Education [50].

For the search and selection of the texts a systematic search of the literature has been carried out following the sequence proposed by Pawson [53]. The first step is to clarify the purpose of the review. In the second step, it aims to search for documents. This stage was divided into three sub-stages: the search for bibliographic references proper, the filtering of the documentation and the synthesis of the documents.

The review was carried out on the main collection of the Web of Science, in documents written in English and published in the last 5 years (2015-19) [48,54,55]. Keywords used in the search are shown in Table 1.

Once this step was finished, the actual hermeneutic work began with the evaluation of the results, the synthesis of the documentation and the creation of the conceptual framework.

**Figure 7:**
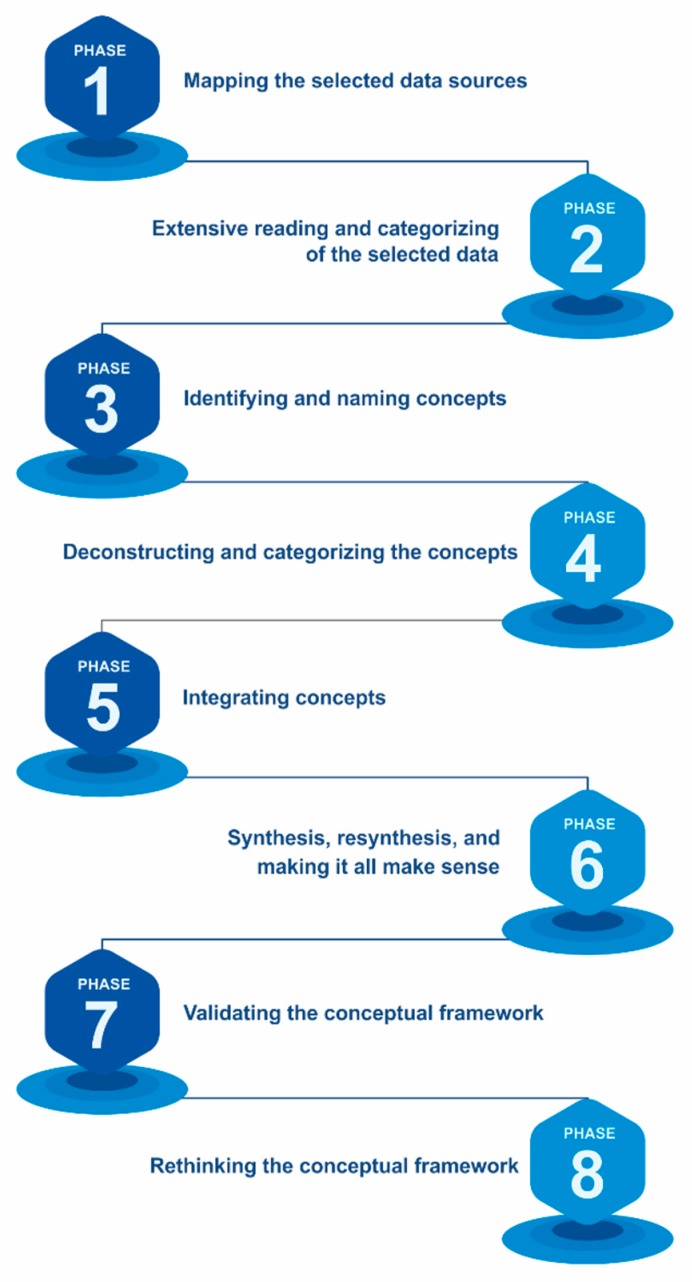
Methodology used to build the proposed conceptual framework [56].

The methodology used to build the proposed conceptual framework consists of the following main phases [56], as shown in Figure 7.

Phase 1: Identification of the selected data sources. The first task is to review the spectrum of multidisciplinary literature regarding the phenomenon in question. This process includes the identification of text types and other data sources.

Phase 2: Detailed reading and categorization of the selected data. The objective in this phase is to categorize the data by discipline and by a scale of importance.

Phase 3: Identification and denomination of concepts. The objective in this phase is to reread the selected data and identify the concepts that group them [57,58].

Phase 4: Build and categorize concepts. The objective of this phase is to find each concept, identify its main attributes, characteristics, assumptions and role. Subsequently it organizes and categorizes the concepts according to their characteristics and ontological, epistemological and methodological role.

Phase 5: Integration of concepts. The objective in this phase is to group conceptions that have similarities in a new concept. This phase dramatically reduces the number of definitions and allows a reasonable number of them to be manipulated.

Phase 6: Synthesis The objective in this phase is to synthesize concepts in a theoretical framework. The researcher must be open, tolerant and flexible with the theorizing process and the emerging new theory. This process is iterative and includes repetitive synthesis until the researcher recognizes a general theoretical framework that makes sense.

Phase 7: Validation of the conceptual framework. The objective in this phase is to test the conceptual framework. The question is whether the proposed framework and its concepts make sense not only for the researcher but also for other academics and professionals.

Phase 8: Rethink the conceptual framework. A theory or theoretical framework that represents a multidisciplinary phenomenon will always be dynamic. It should be reviewed according to new knowledge, comments, literature.

## 3. Results

In this study, it has been detected that the sensorization of industrial plants and the treatment of data through Machine Learning algorithms has not yet been extended enough in the field of sustainability optimization. As a result of this research on the state of the art, five fundamental dimensions are obtained in the approach to these types of problems: applications, data, approaches, tools and sensors. These five aspects will be integrated into a proposed conceptual framework, which aims to be a contribution when facing sustainability engineering projects.

We propose a conceptual framework that may help to understand and situate the research in life cycle analysis using techniques of machine learning, as well as apply the proposed framework to a case study.

**Figure 8:**
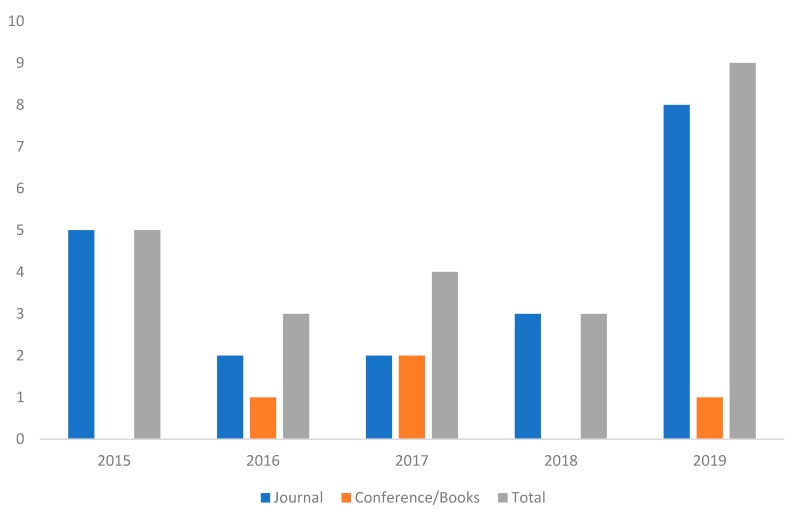
Evolution of the articles published in the last 5 years.

The search result according to the criteria shown in the previous section was 39 documents. Of these documents, 15 were discarded because they were false positives: from the abstract and the title it could be deduced that there was no relation with the topic analyzed in the work. The remaining 24 documents were read in depth. Figure 8 shows the distribution of the documents according to the year of publication and the type of publication (journal or conference proceedings or book chapters).

As can be seen in the figure, most of the 24 documents that have been reviewed correspond to articles published in journals. An increase in the number of publications that address this topic could be inferred from the data, this aspect shows the interest of the work.

From the reading of the previous documents, a total of 11 articles were eliminated because they were outside the scope. For example, for dealing with steam turbine operating conditions [59] or improving the energy use of a farm [60]. Thus, the articles that were finally used for the construction of the framework are shown in Table 2.

As can be inferred from Table 2, only one document 17 meets the four search criteria, one document 13 covers three search criteria. Most of the documents [61,62,63,64,68,70,73] cover two search criteria, the most common being the conjunction of criteria I and II. Only two documents [71,73] meet a single search criterion.

### 3.1. ADAPTS: A Proposal of Conceptual Framework for Engineering Projects

Results have been extracted from the bibliographic study (shown in Table 1). The most relevant characteristics detected in the works analyzed are: (1) the application to which the work refers, (2) the data used, (3) the approach that has been given to the problem addressed, (4) the machine learning tool used and 5) the sensors used or proposed for implementation. These five aspects detected are what form the basis of our conceptual framework. Therefore, the proposed conceptual framework starts from the bibliographic study carried out, which has allowed us to identify the key aspects when addressing the optimization of engineering projects from the perspective of sustainability, using LCA.As previously described in the methodology section, after a detailed reading of the above-mentioned bibliographical references (phase 2 of Figure 7), authors of this article constructed and categorized the concepts (phase 4), integrated the concepts (phase 5) and proposed a pyramid-shaped structure that integrates all the results (phase 6).

The conceptual framework will be validated through a practical application exercise, a case study demonstrating its usefulness (phase 7), shown in the following section. This paper describes a new conceptual framework to be applied in addressing project problems in engineering, from a point of view of sustainability optimization. The conceptual framework provides tools to approach engineering projects in an intelligent and sustainable way. The proposed tools are based on the analysis of the state of the art in the areas of sustainability, life cycle analysis, sensing and machine learning. Five main dimensions have been detected: Applications, Data, APproaches, Tools and Sensors. The results of the “state of the art” study carried out are shown on Table 3.

**Figure 9:**
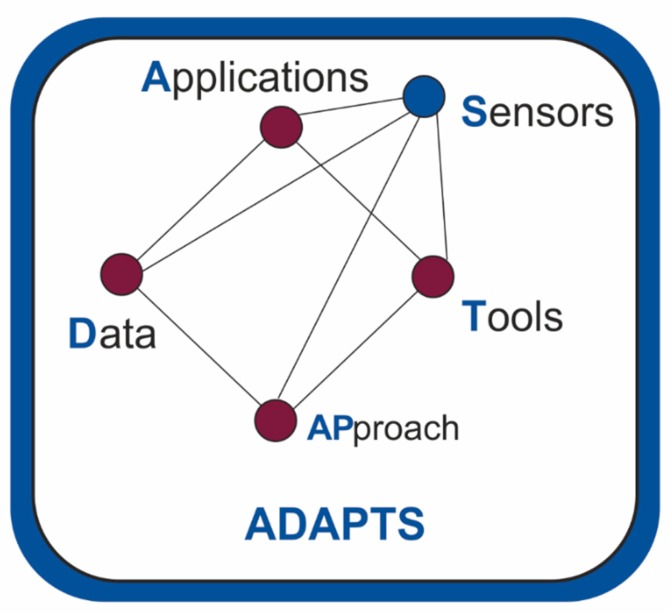
Proposed conceptual framework.

A conceptual framework is proposed that integrates these five dimensions as shown in Figure 9. At the base of this pyramid will be the applications, data, approaches and tools. At the top of the pyramid is the sensorization. As a complement to the proposed conceptual framework, this proposal will be developed for a specific case (a case of a food industry), a development that can be found in the discussion section.

#### 3.1.1. Applications

The first dimension of the proposed conceptual framework is the application of artificial intelligence and machine learning technologies. A first classification of the applications can be referred to the sectors in which they are applied.

Among the 13 articles analyzed; six of them are focused on the construction sector and civil engineering. Traditionally, the construction sector has been quite conservative and resistant to change. However, the emergence of technologies such as Building Information Modelling are causing a change in it. LCA criteria could be used for design structures. This was explored in a review article on the design of concrete structures [66] or in a call for data for the evaluation of structures [69]. Environmental criteria can be used for the design of buildings in the early design stage [71] or for the estimation of total energy consumption from environmental life cycle analysis (LCA) and in cost life cycle analysis (LCC) [68]. In the field of civil engineering, environmental assessment of roads (in its operational phase) was explored by analyzing truck traffic [72] or vehicle speed and road roughness data in Chicago [70].

The design and production of products has been analyzed. For this purpose, LCA was obtained from the data generated in the conceptual design [64] or by analyzing sustainability in manufacturing [65].

Environmental assessment of agricultural activities using artificial intelligence techniques has been analyzed jointly with the energy consumption for the cultivation of sugar cane (planted or ratoon farm) [73] or rice [67].

Finally, some applications consist of using artificial intelligence techniques to address specific aspects of the LCA such as sensitivity to certain factors [70], to asses biological effects using a tool quantitative outcome pathway (qAOP) [63], toxicity characterization of chemical emissions [61] or assess normalization factors in LCA methodology [62].

#### 3.1.2. Data

The data is crucial for the implementation of Machine Learning and Artificial Intelligence methodologies. Data are required for training and making predictions with both supervised and unsupervised techniques.

Obtaining data is not always simple in fact, some authors warn of the need to work in scenarios with little data (austere data environments). This justifies the need for call for data. For example, D’Amico made a request for data geometry, material, type of building and other aspects in the field of structures [69].

In road environmental impact assessment studies, the data came from a company database (Microlise) [72] or from mobility studies in cities such as Chicago [70].

In the field of agriculture, data such as the amount of pesticides, fertilizers, or seeds are needed to determine the environmental impact. This can be done by randomly obtaining data, as was done in [67] to analyze the environmental impact of rice or in [73] for sugarcane farms.

In some investigations, data come from simulations. For example, in Sharif and Hammad [68] extensive data is collected on existing buildings related to several factors including TEC, outside temperature, building envelope components, HVAC and lighting systems; in other occasions the data are obtained using a data generator these data are later validated with experimental data [65].

The conceptual design of products [64] or the early design stage of building [71] are a source of data that can be used in the environmental assessment of products [64] or buildings [65].

Finally, to perform the toxicological characterization of chemical emissions [61], the authors used databases such as USEtox [74], for the normalization of factors [62] the authors used databases such as PPDB, ReciPe.08 or FURs.

#### 3.1.3. Approaches

In the study of the state of the art carried out as part of the process of construction of the proposed conceptual framework, various work approaches have been shown. For example, several of these works [63,66,68,69,71] use the LCA life cycle analysis technique to improve the design. There are also works that focus on the exploitation phase [67,70,72,73], while others focus on the study of the methodology followed in the LCA [61,62,65].

#### 3.1.4. Tools

The use of artificial neural networks is currently a trend in the scientific literature [75,76]. In this area, neural networks have been used to predict energy consumption [68], as a surrogate model to replace simulation software. [69], to obtain LCA results from product characteristics [64], to obtain toxicity with fewer parameters [61], to evaluate energy consumption and environmental impact of agricultural activities [67,73] and to evaluate the energy consumption in order to evaluate environmental impacts of roads [72].

Artificial Neural Networks are often used in combination with other tools such as Simulation-Based Multi-Objective Optimization [68], Bayesian analysis and orthogonal basis polynomial basis system [70], dimensionality reduction techniques and linear regression [61]; adaptive Neuro fuzzy inference system (ANFIS) model and Boruta algorithm [72].

Another similar tool is the Extreme Machine Learning (feedforward neural networks), that together with fuzzy C-means clustering was used to valuate environmental impact in early building design stage. Bayesian Network Models were used too in several applications [63,65]. Linear regression was used to estimate normalization LCA factors [62].

#### 3.1.5. Sensors

Throughout this study the enormous relevance of using sensing when addressing an objective of intelligent sustainability in engineering projects has emerged.

This sensorization is essential to be able to train models, validate and generalize them. It is also useful to have intelligent sensors that allow us to have control of the data in the exploitation process.

Several of the works analyzed use simulation data, data taken from databases or public data. For example, [63] works with toxicity data, in [71] it deals with properties of the buildings or in [70] traffic data is used. The enormous utility of having data from sensorization is evident.

### 3.2. Case Study

A new conceptual framework has been described that could be applied to address the problems of engineering projects, from the point of view of sustainability optimization. The proposed conceptual framework integrates five dimensions: applications, data, approaches, tools and sensing. This conceptual framework is developed below for a specific case (olive oil sector), showing its applicability and usefulness.

The case study intends to answer the five questions that constitute the validation phase of the conceptual framework [77].

**Figure 10:**
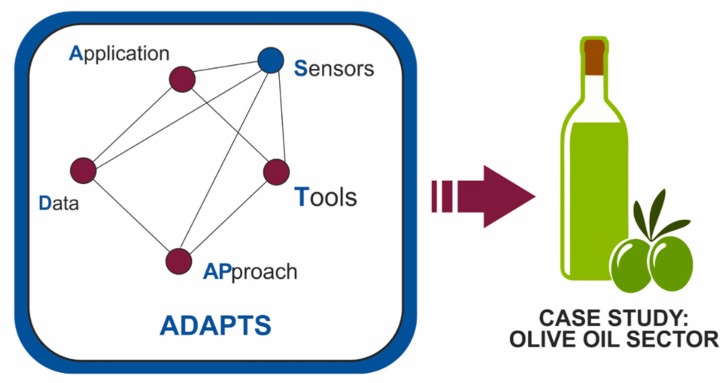
Conceptual framework. Study case.

(1) Is this framework useful? [78,79].
(2) Does it provide a common language from which to describe the situation under scrutiny and to report the findings about it [79].
(3) Does it develop a set of guiding principles against which judgments and predictions might be made?
(4) Does it act as a series of reference points from which to locate the research questions within contemporary theorizing?
  Does it provided a structure within which to organize the content of the research and to frame conclusions within the context? [78].
(5) Below is an application of the proposed conceptual framework to a specific case study, the olive oil sector. Each of the dimensions of the ADAPTS pyramid will be discussed in this example (Figure 10).

**Figure 11:**
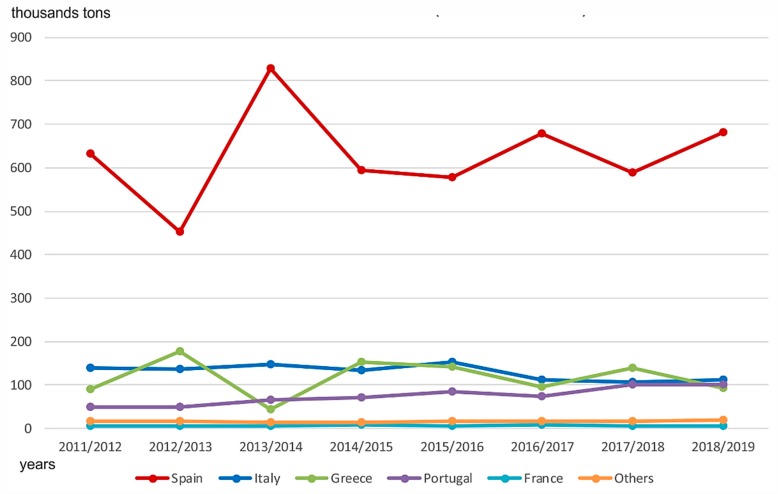
Total exports volume. Compiled by the authors based on [81].

Olive oil production is a very important activity in the Mediterranean area. Oil processing influences the environment by causing resource depletion, land degradation, air emissions and waste generation [80]. Figure 11 illustrates the total volume of olive oil exports within the European Union, by country.

Different phases must be considered to carry out the LCA of oil production. Firstly, in the agricultural phase, the cultivation of the olive trees is considered, as well as the pruning and harvesting. In the phase of production, oil extraction must be analyzed. Some studies also consider aspects such as packaging, distribution, consumption and transport. Finally, the management of the residues must be considered. There is a consensus in the scientific literature that the phase that has the greatest impact is agriculture. This is due to the use of fertilizers, phytosanitary products and irrigation [82].

Thus, it is necessary to consider that the processes are compatible with both environmental protection and efficiency, throughout the entire product life cycle. This extends to the handling, packaging and labeling of products. It is necessary to take into account the reduction of inputs such as fertilizers, phytosanitary products or fossil fuels. The development of technologies is sought to optimize the use of valuable water resources in an environment with water scarcity. It is advisable to use climate monitoring stations through olive groves, making extensive use of soil moisture sensors, salinity sensors and reporting systems to ensure that water is used for maximum efficiency. Carbon emissions play an important role in atmospheric conditions and climate change, so it is necessary to reduce emissions. It is also necessary to reduce the energy use of the electricity grid, seeking to reduce the carbon footprint.

#### 3.2.1. Application

The olive trees produce the olive that is transported to the oil mills, where after a few mechanical processes the virgin olive oil is extracted. This oil is packaged directly in packaging machines belonging to oil mills or in independent packaging machines, in the case of extra virgin olive oil. If not, it is sold to refineries where refined olive oil is obtained. From the mixture of extra virgin olive oil and the refined olive oil is obtained which will be packaged in the refineries. Oil distribution can be done through distribution platforms, hypermarkets, supermarkets and traditional stores [83], being able to start in olive crops with different forms of exploitation.

There are three cultivation modalities: traditional or extensive, intensive and superintensive cultivation. The first cultivation modality is the usual one in the areas of olive tradition; they can be irrigated or dry. Its planting density is around 80–120 trees/ha, with one or several feet. This makes the collection is mostly done manually through the help of machinery. In the intensive cultivation system, always in better soils and irrigated land, we work with a planting density of 200–400 trees/ha. The collection systems are very similar to those of the traditional olive grove. In the superintensive (or "in hedge") the planting density is more than 800 trees/ha. These systems constitute what has come to be called "new olive growing" [83]. The olive harvest in this type of plantation is fully mechanized thanks to the existence of machines designed for this type of olive grove. The impact of this first stage is the highest according to most studies [82].

Once the olive has been collected using the methods required for each type of crop (traditional, intensive and extensive), the industrialization process begins. To do this, the olive is cleaned well on the farm itself where it is collected or, it is taken to the mills and there it will undergo the cleaning process. In the oil mills, the olives caught in flight are separated, that is, from the tree, or from the ground since, the quality of the oil varies depending on the origin and possible damage that it may have suffered. Once separated, the oil extraction process begins by grinding, shaking, horizontal and vertical centrifugation and decanting. Once these phases are finished, the oil is stored until the packaging process begins, which can be done in the mills themselves, if they have the capacity to do so, or in independent packaging machines. A third option is packing machines linked to refineries where the lampante oil is taken to undergo a neutralization process. This oil is the result of a last centrifugation which makes it very acidic and not suitable for human consumption. It is called that because it was formerly used for the combustion of oil lamps.

Olive oil packaging must go through the filling and capping, labeling and packaging phases. The distribution of olive oil can be carried out on different supports depending on what will be your final recipient. The bulk distribution involves selling the unpacked oil, so that the buyer is responsible for its bottling and subsequent distribution. Packaged oil, in the mills themselves or in independent packaging machines, is usually sold directly to the final consumer or packed to small businesses. Finally, pallet oil is sold wholesale to new forms of commercial distribution such as supermarkets and hypermarkets.

In the last ten years, the olive oil value chain has benefited from innovations introduced with the idea of making oil more efficient and profitable. The most significant innovations have been exposed in the work, helping us to do so with the specific value chain mentioned above [84].

#### 3.2.2. Data

One of the main difficulties in carrying out an adequate LCA of oil production is the need to have public databases with information about all stages of the process [82].

In this way, information about soil salinity and climatic characteristics can be crucial to develop an optimization of the impact of this activity. This information could be collected by sensors.

The main technological variables that influence the elaboration process are:

(1) *Degree of grinding*. The degree of grinding indicates the average size in which the hardest parts of the paste remain. Grinding too thick means a weak breakage of the tissues that leads to a decrease in exhaustion. On the other hand, too fine grinding causes a greater increase in the temperature of the paste that has a negative impact on the quality of the oil, and can generate more emulsions in the pasta that deplete exhaustion. Additionally, too fine grinding causes problems of bindings in the mill and increases energy consumption [85].
(2) *Temperature and beat time*. These parameters are, perhaps, the most decisive in the quality of the oil to be obtained. The increase in the temperature of the pasta in the blender reduces its viscosity, which favors the aggregation of the drops of oil and therefore improves extraction performance. On the other hand, the increase in shaking time also favors the change in the structure of the paste that allows increasing the depletion of the pomace. However, both parameters have a negative impact on the quality of the oil obtained, since the increase in temperature accelerates the speed of the reactions that take place in the blender and favors the loss of volatile components.
(3) *Composition and structure of the pasta at the exit of the blender*. In the shaking of the so-called difficult pastes, which are those pastes that present difficulties in extracting the oil, it is necessary to use adjuvants (natural microtalco and, where appropriate, water) to improve their behavior. A repair of the deficient pulp entails substantial increases in the oil contained in the pomace, while the addition of adjuvants has no influence on the quality of the oils obtained [86].
(4) *Degree of moisture of the paste in decanter*. This parameter has great relevance in depletion, since it will determine the thickness of the rings inside the decanter and, therefore, the operating conditions of the machine.
(5) *Residence time in decanter*. The residence time in the decanter is determined by the established production rate, which is generally imposed by the olive oil entering the mill and the capacity of the installation. Operating the decanter at higher rates than recommended implies a significant loss of fat in the pomace.
(6) *Parameters of the operation within the decanter*. The differential screw-bowl speed and the discharge height of the oil phase determine the width of the different rings within the decanter [87]. A correct choice of these parameters makes it possible to improve depletion without influencing the quality of the oil obtained.
(7) *Parameters specific to the operation of the vertical centrifuge*. The temperature of the addition water must be adjusted to the temperature of the oil so as not to affect its organoleptic properties and avoid the formation of emulsions that induce the loss of oil with the wash waters. Likewise, the water flow must be adjusted to that of oil for the proper functioning of the machine in terms of losses. Finally, the discharge frequency of the cumulative strips is an important parameter since it influences the quality of the oil obtained and the oil losses in the operation.
(8) *Parameters of the decantation in stainless steel tanks*. The main parameter is the residence time of the oil in the tanks and the frequency of the purges of the erasures. A short residence time means that the oil remains with a high level of moisture and impurities, while a residence time that is too high and a poor purge frequency can be supposed to damage its organoleptic characteristics [88].

These variables are related to the quality of the olive oil, but also to other aspects such as the energy consumption or the waste generated in the process. In this way, with a suitable sensing, the impact that the activity has on the environment can be optimized.

#### 3.2.3. Approach

As a result of the growth of the food and beverage industries, a particularly demanding sector with production management is found. Economic behavior (rigid supply, inelastic demand), administrative intervention in primary productions, food safety requirements, diversity of products, variability of productions, etc. to which we must add those that also affect the rest of the economy: financial fluctuations, acceleration of applicable technologies, globalization of markets, etc. [89].

The agro-food industry is a very important strategic sector for our economy. It generates more than half a million direct jobs, above the total manufacturing industry and the Spanish economy as a whole [90]. For this reason, the case study has been focused on this sector, due to the great potential. These companies show and the possibilities they provide. Thanks to the new Industry 4.0 approach, making use of information technology, it is now easier to convert data into useful information for decision making.

Tools such as data mining and predictive techniques organizations have information that helps them raise more precise, effective and applicable business strategies in shorter periods of time. Before they can interpret the process data, companies must treat them to reduce the problem to be treated and optimize the available resources. Data mining provides the means for the treatment of productive data.

On the other hand, there are different predictive techniques, each with its benefits and deficiencies, which provide a new way for companies to make decisions. The Spanish agri-food industry is likely to benefit from the Industry 4.0 approach. In a globalized market, any small advantage over the competition can make a big difference. As the food and beverage market is very demanding regarding production management, the tools discussed above offer organizations a competitive advantage to value [91].

#### 3.2.4. Tools

As shown in the section dedicated to tools, the use of artificial neural networks is currently a growing trend in recent academic literature [75,76]. In the field of sustainability applied to engineering projects, neural networks have been used to predict energy consumption [68], as a surrogate model to replace simulation software. [69], to obtain LCA results from product characteristics [64], to obtain toxicity with fewer parameters [61], to evaluate energy consumption and environmental impact of agricultural activities [67,73] and to evaluate the energy consumption in order to evaluate environmental impacts of roads [72], among many other applications.

Artificial Neural Networks are often used in combination with other tools such as Simulation-Based Multi-Objective Optimization [68], Bayesian analysis and orthogonal basis polynomial basis system [70], dimensionality reduction techniques and linear regression [61]; adaptive Neuro fuzzy inference system (ANFIS) model and Boruta algorithm [72].

In this case of application it has been decided to use ANN as a tool due to the reasons already stated: because the use of artificial neural networks is currently a growing trend, because in the field of sustainability applied to engineering projects ANN have been used in numerous applications and because ANNs are often used in combination with other tools.

Artificial neural networks (ANN) are a machine learning tool that can be useful for predicting one or more variables in complex systems. ANNs consist of an input layer, a variable number of hidden layer(s), an output layer, weights and connection biases, an activation function and an addition node. Each neuron constitutes a computational unit, integrated in a layer. Each layer takes as input values those calculated in the previous layer and generates an output value for the next layer. The input layer provides the input values of the network, which are fed to the hidden layer. Each hidden layer consists of several neurons that calculate an output using all the inputs of the input layer and a predefined set of weights and biases. During the learning process, each neuron calculates a single output value based on its input data from the previous layer. This result can be fed to the next hidden layer or to the output layer. The output layer takes as inputs all outputs of the last hidden layer and produces the final output of the ANN [92].

#### 3.2.5. Sensors

As we have mentioned before, in order to perform an adequate LCA of the oil manufacturing process it is essential to have a reliable database about the variables that influence the process. In this sense, a correct sensor network arranged throughout the process can contribute to improve the LCA and with it, the process optimization can be carried out crucial aspect for the optimization of the LCA of olive oil is the cultivation phase. In this respect, Previous studies have shown that sensing can help optimize olives drip irrigation by controlling soil moisture and knowing the weather using a weather station [93,94]. LED Scanner Sensor have been used for measuring olive oil canopies [95].

One of the problems that arise in the automatic control of oil mills is the difficulty of having the necessary information about the process. In fact, in order to adapt the operating conditions of the plant from a global point of view, it would be necessary to know the characteristics of the input fruit, the characteristics of the pasta in the blender, the composition and flow of the flows of entry and exit of the decanter, etc. To control the mill with the objective of maximizing quality, it would be necessary to be able to measure this variable, which in general would be a combination of the values of different chemical and organoleptic parameters.

Many of these variables are qualitative and difficult to measure online, so the use of indirect measures or the use of sensory fusion techniques are intuited as necessary alternatives to be able to estimate the values of the parameters. An important sensor technology for the automatic control of oil mills are Near Infrared Spectrocopy (NIR) sensors, since this technique allows the construction of sensors to estimate the moisture and fat content of the pulp and pomace. In addition, it allows characterizing the oil obtained from the process in terms of quality by estimating a series of chemical parameters, such as acidity, peroxide index, K270 and total polyphenol content [96].

The use of this type of sensors together with neural networks it allows to improve data obtained from this type of sensors [97,98]., which together with the possibility of using these sensors for measurements online makes them fundamental tools for automatic control of the oil mill. Another technology of relevance sensors is the arrays of voltmetric sensors, which allow evaluating the polyphenol content of oil [99]. The construction of sensors in line with this technology can provide very valuable information for the control of oil mills in order to maximize the quality of the oils obtained, since they allow information on parameters sensitive to the elaboration process and very related to the oil quality In this regard, they have been applied this same type of sensors to monitor online the accumulation of volatile components in the mixer [100], which opens the doors to its use for automatic control.

The first reference of using neural networks to building a virtual sensor is located in [101], where a neural network is designed and implemented to infer depletion and moisture from the alpeorujo. The use of neural networks to infer characteristics of the oil produced from characteristics of the input fruit and the process parameters can be consulted in [102], as well as the use of artificial vision to capture information from the input olives, in this case the index of maturity. In the same line of behavior prediction of the installation from neural networks, build a neural network to predict the depletion of the pomace from variables characteristic of the fruit (fat and moisture content) and technological variables such as the beat temperature, the addition of micro-total, paste inlet flow into the decanter, paste moisture and oil outlet height from the decanter [103]. These works present the bases for the construction of virtual sensors that allow variables to be included in control loops that otherwise would not be possible to measure online [88].

**Figure 12:**
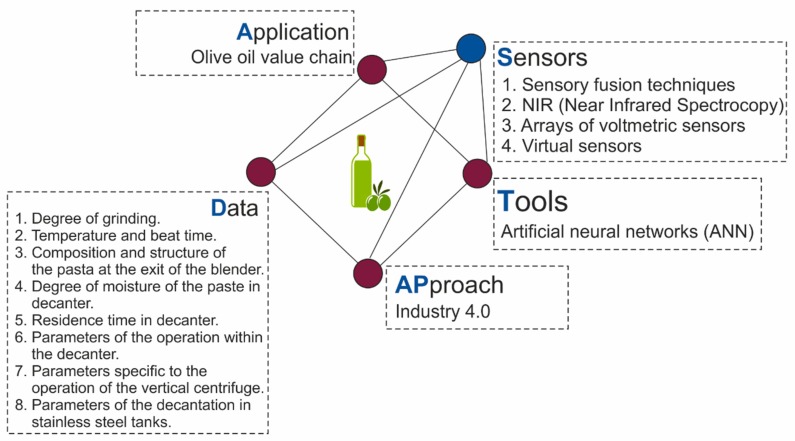
Conceptual framework. Study case.

The Figure 12 shows the result of applying the proposed conceptual framework to the case study.

The case study is a validation of the conceptual framework proposed in Section 3.1. The conceptual framework is a useful tool to analyze the production of olive oil from an environmental perspective. It allows to create a common language for the analysis of the LCA using machine learning tools.

**Figure 13:**
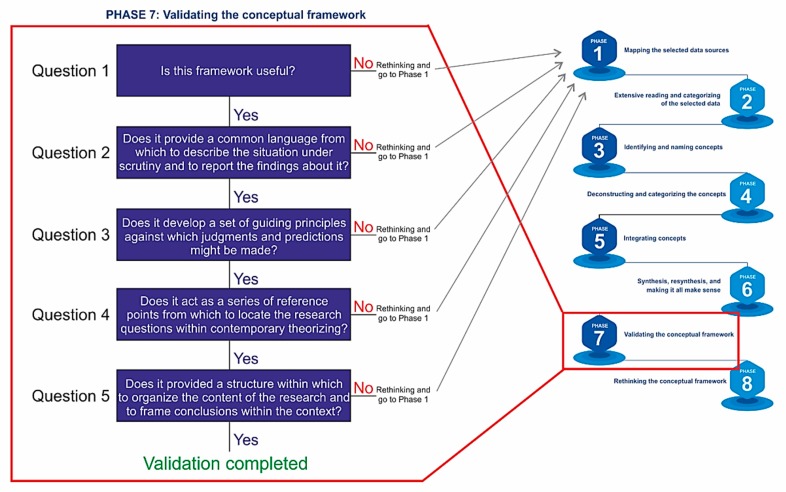
Validation of the Conceptual Framework.

The framework can be a reference point that will allow to guide future research and to organize the content of future investigations. In this way, it can be stated that the conceptual framework answers the questions proposed by Smith [77] (see Figure 13).

Rykiel [104] argues that models can be validated pragmatically, while theoretical validity is always provisional. In this regard, he, like Matalas et al. [105], distinguishes between theoretical and conceptual frameworks. According to Rykiel [104], validation is not a procedure to test the conceptual framework, but rather a proof of its suitability for its intended use. The domain of applicability (of the conceptual model) will be the conditions for which the conceptual model has been tested, that is, compared to reality as far as possible and considered suitable for use [106].While a theoretical framework is used to test theories, to predict and control the situations within the context of a research inquiry, a conceptual framework is aimed at development of a theory that would be useful to practitioners in the field [107]. In this sense, this case study remarks that the proposed conceptual framework develops a set of guiding principles against which judgments and predictions might be made. Specifically, the conceptual framework proposes to focus the study on five axes: the application, the data, the approach, the machine learning tools to be used and the necessary sensing.

In the case studied, using these guiding principles proposed, it is concluded that for the application in the case of olive oil it is useful to have data related to the quality of the olive oil, but also to other aspects such as the energy consumption or the waste generated in the process. It is also claimed that the agri-food industry is likely to benefit from the Industry 4.0 approach, and it is proposed that use of ANN In this case of application it has been decided to use ANN as a tool, and address the need for adequate sensing, because to perform an adequate LCA of the oil manufacturing process it is essential to have a reliable database about the variables that influence the process. In this sense, a correct sensor network arranged throughout the process can contribute to improve the LCA and with it, the process optimization can be carried out crucial aspect for the optimization of the LCA of olive oil is the cultivation phase.

The results obtained from the bibliographic study (shown in Table 3) provide the most relevant characteristics detected in the analyzed works: 1) the application to which the work refers, 2) the data used, 3) the approach that has been given to the problem addressed, 4) the machine learning tool used and 5) the sensors used or proposed for implementation. These five aspects detected are those that form our conceptual framework. Therefore, the proposed conceptual framework starts from the bibliographic study carried out, which has allowed us to identify the key aspects when addressing the optimization of engineering projects from the perspective of sustainability, using LCA. As far as we know, industrial plant sensing and data processing through Machine Learning algorithms has not yet been extended sufficiently in the field of sustainability optimization for engineering projects. The way to bridge this opportunity gap detected in the investigation of the state of the art (Figure 5) constitutes our research question, for which the proposed conceptual framework aims to constitute a reference point that helps to locate the research question within the contemporary theorizing.

## 4. Discussion

The main objective of this work is the development of a conceptual framework for the integration of artificial intelligence and life cycle assessment (LCA). In the literature review that has been carried out, it has been detected that the sensorization of industrial plants and the treatment of data through Machine Learning algorithms has not yet been extended enough in the field of sustainability optimization.

This work has focused on the evaluation of sustainability using the LCA technique, because it uses some indicators that allow quantifying environmental impacts.

As far as we know, industrial plant sensorization and data processing through machine learning algorithms have not yet been extended enough in the field of sustainability optimization. An opportunity gap has been detected in the investigation of the state of the art.

As shown in Figure 5, our work is part of the triple coincidence between the sectors of sustainability (especially through life cycle analysis), sensorization and the application of machine learning techniques. In the bibliographic study conducted, the confluence between machine learning tools and the life cycle analysis technique has been enhanced, and the results obtained are shown in the corresponding section. It has not deepened the binomials machine learning-sensors and sensors-LCA, because the searches of these binomials in Web Of science give a huge number of results, some of them not very relevant, because they are very closely related terms.

In Pérez et al. [108], sustainability is taken into account from the stage of conceptualization and design of an engineering project. This paper presents a review of the state of knowledge and project design methodology to obtain a first comprehensive approach and an initial structure of a generic nature, as the first step to provide a practical way to facilitate analysis and application of sustainability criteria in the design of an engineering project.

In García et al. [109] a model is shown, in consecutive stages, for sustainability analysis: definition, interpretation, conception of goals and specifications, measurement and evaluation. Specifically, these are principles that integrate a philosophy of sustainable design around innovation and creativity in engineering in general, and in chemical engineering in specific.

Vanegas et al. [110] proposes a model to incorporate sustainability criteria and principles in the design, construction and management of infrastructures, which he proposes applicable to any sustainability discussion in engineering, architecture and construction. Its model encompasses three visions of sustainability (a global vision; a sector vision and a project vision); three maps for the implementation of sustainability (strategic, tactical and operational) and the indication of sustainability principles from specific sources, manifesting a freedom in their adoption as long as these principles can be made effective by expressing them in terms of specific goals; quantifiable objectives associated with the goal; and an application plan, for specific projects.

The sustainability evaluation model of engineering projects using specific sustainability criteria proposed by Segalas et al. [111], associates measurement and evaluation variables in the different stages of the life cycle of a project; and a learning assessment model that engineering students acquire in different subjects through the specific use of concept maps.

Vezzoli et al. [112] propose the use of guides and checklists for the design of a certain type of product with an eco-efficient approach, pointing out the importance of developing specific guides and checklist for each type of product, as tools to realize the design sustainable of an object.

In Labus et al. [113], the application of sustainability indicators in the design process of an engineering project is proposed. Based on the analysis of current computer frameworks, it establishes a framework of indicators that can guide the different stages of a project, a product or a system; to link sustainability principles throughout the project life cycle and between the different stages that comprise it. It also brings the qualitative importance of sustainability assessment with decision analysis techniques.

Other elements of consideration from a project management point of view are provided in Fernandez et al. [114], to identify sustainability factors and indicators in engineering projects in general and civil engineering projects in particular.

The results of the study conducted in Armenia et al. [115], indicate that the academic literature on this subject is still in diapers, but that the attention of academics is growing and opens new directions of research. Based on the results of the literature review, a new conceptual framework is proposed that links five key dimensions of sustainable project management: corporate policies and practices, resource management, life cycle guidance, stakeholder participation and organizational learning.

In none of the existing conceptual frameworks, as far as the authors know, a conceptual framework is proposed that integrates the aspects of sustainability through the application of Machine Learning techniques in engineering projects.

A conceptual framework is proposed that can help in the approach to these types of problems. This paper has described a new conceptual framework that could be applied to address the problems of engineering projects, from the point of view of optimization of sustainability.

In a research work not only influences the choice of the topic and the approach of the problem, but also affects the selection made of the research procedures, the underlying theories that explain the topic of interest, and the specific way in which results are analyzed and disseminated.

When carrying out the work, the researchers incorporate and make initial formulations of the research problem, which should be based on the empirical evidence that best supports the existing theoretical perspective(s). It is convenient to develop the research project based on a conceptual framework, related to the subject in question, which makes reference to the explanations given for the research problem of interest, the most appropriate procedures to answer the research questions, as well as the strength of the evidence achieved in terms of methodological instrumentation.

The initial plan for the development of a framework that supports the research to be carried out, includes not only the assumptions from which the research starts, but also shapes the way in which the data is collected, which in turn determines or establishes the limits of the kinds of analysis that can be used.

Certain techniques are more compatible with some assumptions than with others, which means that at the time of selecting a series of research methods, a certain theoretical position is necessarily assumed.

The scientific method in general, favors the scope of objective knowledge, has a basic methodology that uses research logic and research procedures, and is invariable regardless of the kind of data studied. On the other hand, the researcher can observe, relate and make sense of the events he/she can remember, imagine, compare, differentiate, integrate, and thereby place them in his/her proper perspective. And part of other assumptions too, it has the means to create instruments that extend these capabilities or reduce their restrictions. In a broad sense, scientific theory refers to a series of logically interrelated propositions or assertions that empirically make sense, as well as the assumptions the researcher makes.

The conceptual framework is actually a bibliographic investigation that talks about the variables that will be studied in the research, or the relationship between them, described in similar or previous studies. It refers to perspectives or approaches used in related studies, its goodness or property and its relevance to the current study is analyzed.

More specifically, it leads to the establishment of hypotheses, suggests ways of analysis, or new perspectives to be considered, and at the same time, helps interpret the results of the study [116].

The conceptual framework proposed in this work provides tools to address engineering projects in an intelligent and sustainable way. The proposed tools are based on the analysis of the state of the art in the areas of sustainability, life cycle analysis, detection and machine learning.

A conceptual framework that integrates five dimensions is proposed. At the base of the proposed pyramid will be the applications, data, approaches and tools. At the top of the pyramid is the sensorization. As a complement to the proposed conceptual framework, this proposal has been developed for a specific case (olive oil sector), showing its applicability and usefulness.

## 5. Conclusions

This paper proposes a conceptual framework applicable to optimization problems under sustainability criteria in engineering projects, making use of current machine learning techniques.

A systematic literature review has been carried out. From the selected documents, the texts were analyzed, and the conceptual framework was proposed. A graphic representation is also proposed to clearly define the variables of the proposed conceptual framework and their relationships.

The proposed conceptual framework consists of five dimensions. At the base are: (1) the application to which it is intended, (2) the available data, (3) the approach and (4) the tool used. At the top of the pyramid, (5) the necessary sensing.

The first dimension of the proposed conceptual framework is the application to which the conceptual framework is applied. A first classification of applications may refer to the sectors in which they are applied.

Data is crucial for the implementation of machine learning and artificial intelligence methodologies. Data is necessary to train supervised algorithms and make predictions in unsupervised algorithms.

In the study of the state of the art carried out as part of the process of construction of the proposed conceptual framework, various work approaches have been shown.

Another very important dimension of the proposed framework is the artificial intelligence and machine learning tools used.

Throughout this study, the enormous relevance of using sensing when addressing an objective of intelligent sustainability in engineering projects has emerged. This sensorization is essential to be able to train models, validate them and generalize them. It is also useful to have intelligent sensors that allow us to have control of the data in the exploitation process.

This work is part of a broader line of research, in terms of optimization under sustainability criteria. We hope that the proposed framework will serve as a basis for future research related to this topic.

## Figures and Tables

**Table 1:** Keywords used during systematic literature review.

Keywords	Search Criteria
“Machine Learning” and “LCA”	I
“Machine Learning” and “Life Cycle Assessment”	II
“Artificial Intelligence" and “Life Cycle Assessment”	III
“Artificial Intelligence” and “LCA”	IV

**Table 2:** Documents used to construct the conceptual framework.

ID [ref]	Tittle	Year	Search Criteria			
			I	II	III	IV
A [61]	Machine learning for toxicity characterization of organic chemical emissions using USEtox database: Learning the structure of the input space	2015	1		1	
B [62]	Extending life cycle assessment normalization factors and use of machine learning - A Slovenian case study	2015	1		1	
C [63]	Limitations of toxicity characterization in life cycle assessment: Can adverse outcome pathways provide a new foundation?	2016	1		1	
D [64]	Quantifying the impact of sustainable product design decisions in the early design phase trough machine learning	2016	1		1	
E [65]	A Data-Driven Approach for Improving Sustainability Assessment in Advanced Manufacturing	2017	1		1	1
F [66]	Computational design optimization of concrete mixtures: A review	2018		1		
G [67]	Integration of artificial intelligence methods and life cycle assessment to predict energy output and environmental impacts of paddy production	2018			1	1
H [68]	Developing surrogate ANN for selecting near-optimal building energy renovation methods considering energy consumption, LCC and LCA	2019	1	1		
I [69]	Machine Learning for Sustainable Structures: A Call for Data	2019	1	1	1	1
J [70]	Model uncertainty analysis using data analytics for life-cycle assessment (LCA) applications	2019	1		1	
K [71]	Assessing environmental performance in early building design stage: An integrated parametric design and machine learning method	2019		1		
L [72]	A machine learning approach for the estimation of fuel consumption related to road pavement rolling resistance for large fleets of trucks	2019		1		
M [73]	Combined life cycle assessment and artificial intelligence for prediction of output energy and environmental impacts of sugarcane production	2019			1	1

**Table 3:** Results for State of the art.

ID	Application	Data	Approach	Tools	Sensors
A [61]	Predict energy consumption of buildings	TRNSYS simulation data. Data collected on existing buildings.	Energy. Performance prediction	ANN + SBMO (genetic algorithms)	Literature review
B [62]	Reduce the impact of Structures	Geometry, material, building type and other key parameters	A more sustainable built environment	Machine Learning + Artificial Neural Networks	Resource efficient built environment lab
C [63]	Predict the sources of uncertainty in the LCA: Chicago Pavement LCA	Traffic data report	Objective uncertainty quantification (UQ)	Various data analytics methods were used to conduct a thorough model uncertainty analysis	Traffic, Speed IRI Collected from 2015 Traffic data report
D [64]	Sustainability in manufacture	Manufacture data using simulation models	Data-driven modeling	Data-model-decision network	Cheaper monitoring tools and pervasive wireless technology enables environmental data to be collected. Manufacturing process data is most often proprietary
E [65]	To asses biological effects using a tool quantitative outcome pathway (qAOP)	Toxicity data. The emission levels. Data of inventory analysis	Toxicological LCIA models and assumptions	Mechanistic; Probabilistic supervised machine learning models; and Weight of evidence	Experimental toxicity data
F [66]	Estimate LCA results from product properties. 37 case study	LCA data. Data generated during conceptual design.	Guidelines, Heuristics, Standards Methods Preferences	Multi-layer perception neural network with back propagation training	Product attributes
G [67]	Toxicity characterization of chemical emissions in Life Cycle Assessment (LCA)	Properties of the chemical compounds being assessed (databases) buildings.	Usetox model	Dimensionality reduction techniques.	Environmental properties
H [68]	LCA normalization factors	A pesticides properties database PPDB, ReciPe .08 and FURS.	LCA normalization	Linear regression using Java program Package	Environmental indicators
I [69]	Estimate LC environmental impacts and output energy of sugarcane production (planted or ratoon farms)	Ecoinvent 2.2 databases	Cradle to grave approach	Artificial neural networks (ANNs) and adaptive neuro fuzzy inference system (ANFIS) model	Used resources emissions, Used electricity
J [70]	Estimate LC and energy of paddy production	Agricultural input parameters from 240 paddy producers.	CExD cumulative. Energy Demand	Artificial neural networks (ANNs) and adaptive neuro fuzzy inference system (ANFIS)	Paddy production process
K [71]	Evaluate environmental impact in early building design stage	Generated samples	Parametric design	Fuzzy C-means clustering and extreme learning machine	Properties of the building
L [72]	Energy consumption of the trucks to evaluate the operation phase of the pavements.	Database of Micrilise Ltd. Road geometry and condition of the road surface for each vehicle in the databases	Enveloping methods (Boruta)	Boruta algorithm and Neural Networks	Standard sensors (SAE International 2016) that keep track of various parameters (including consumption)
M [73]	Design and optimization of concrete structures	Literature review	Optimization Decision Making	ANNs, instance-based learning, decision-trees and SVMs.	Concrete mixture parameters
